# The proliferation of derivative and redundant studies in endocrinology due to the application of Mendelian Randomisation and other methods to open databases

**DOI:** 10.3389/fendo.2024.1400583

**Published:** 2024-06-10

**Authors:** Jonathan H. Tobias, Katherine Samaras, Richard Ivell, Terry F. Davies, Åke Sjöholm, Iwan Day-Haynes, Jeff M.P. Holly

**Affiliations:** ^1^Musculoskeletal Research Unit, Translational Health Sciences, Bristol Medical School, University of Bristol, Bristol, United Kingdom; ^2^MRC Integrative Epidemiology Unit, Population Health Sciences, Bristol Medical School, University of Bristol, Bristol, United Kingdom; ^3^School of Clinical Medicine, Faculty of Medicine and Health, University of New South Wales, Sydney, NSW, Australia; ^4^Department of Endocrinology, St Vincent’s Hospital, Darlinghurst, NSW, Australia; ^5^Clinical Obesity, Nutrition, and Adipose Biology Lab, Clinical Science Pillar, Garvan Institute of Medical Research, Darlinghurst, NSW, Australia; ^6^School of Biosciences, University of Nottingham, Sutton Bonington, United Kingdom; ^7^Thyroid Research Unit, Department of Medicine, Icahn School of Medicine at Mount Sinai and James J. Peters VA Medical Center, New York, NY, United States; ^8^Department of Internal Medicine, Gävle Hospital, University of Gävle, Gävle, Sweden; ^9^Publishing Development, Frontiers in Endocrinology, Frontiers Media SA, Lausanne, Switzerland; ^10^Faculty of Medicine, School of Translational Health Science, Bristol Medical School, University of Bristol, Southmead Hospital, Bristol, United Kingdom

**Keywords:** Mendelian Randomisation, meta-analysis, endocrinology, bibliometric analyses, epidemiology

One of the challenges for all scientists and clinicians is staying abreast of the increasingly vast literature which advances medical science and influences clinical practice. Publications in the life sciences are growing at the rate of 5.1% per year with a doubling time of 14 years (1). Whilst on the one hand, this represents advances in the life sciences, within this increase there has been a profusion of studies that are based on data, observations, and/or concepts that have not been generated by the authors but are derived from publicly available sources. These have recently been growing at a much faster rate. In comparison to the general growth rate in published life sciences manuscripts of 5.1% per year, according to the publications listed on PubMed over the last decade the average yearly increase in meta-analyses was 26.3%, in bibliometric analyses was 35.2% and Mendelian Randomisation analyses was 147%.

Reviews, meta-analyses and bibliometric studies are all mechanisms to synthesise, coalesce or integrate evidence. Putting pieces of a jigsaw together to glimpse the bigger picture. With the deluge of individual publications across the literature such syntheses of the evidence regarding a specific topic can be extremely useful. Within endocrinology a quality review aims to clarify how a hormone may be produced or operate in physiology or a specific pathology. Reviews will often take evidence from experimental models, both *in vitro* and animal models, together with human clinical studies to integrate these to consolidate a paradigm for hormone action. These are of value if significantly more evidence has appeared since the last synthesis or if some distinct insight is applied to put the pieces together in a slightly different way to create a different picture, get across a new concept or create a new hypothesis that may stimulate further work to acquire more pieces of the jigsaw. However, if the same pieces are collected together to produce the same picture then this will be of little interest to those in the field who will already know this information and no value to those from outside the field who wish to learn as the information is already available. Many bibliometric studies lack any insightful evaluation of the evidence covered or how it is synthesised. More concerning are syntheses that lack rigour and promote false concepts and hypotheses. This can occur if the selection of reports and/or of data that are included in a synthesis is biased or does not adequately account for the quality aspect of the findings included. There are many other sources of bias that can occur, such as the bias for just publishing positive and significant findings.

Unfortunately, many published syntheses are flawed. Evaluations of systematic reviews and meta-analyses published across biomedical research revealed many to be poorly conducted and conclusions drawn either redundant or misleading (2, 3). The redundancy in some fields can be considerable with up to 20 meta-analyses of the same topic (2). The increase in published meta-analyses, particularly those emanating from China, was reported previously in 2013 (4). In 2013, PubMed listed 13,239 published meta-analyses; by 2023 there were 34,905 listed, indicating that this trend for generating meta-analyses continues upwards. At Frontiers in Endocrinology we received 341 submissions of meta-analyses last year, up from 73 in 2020, with 71.3% of these coming from China. Last year our journal also received 190 submissions describing bibliometric studies, up from 3 in 2020, prompting a clear instruction to authors that such studies will no longer be considered. More open science has provided increasing opportunities for secondary data use, especially in the case of larger data collections that are representative of the wider population, with detailed meta data facilitating use by external research groups. This has led to a proliferation of studies based on datasets such as the National Health and Nutrition Examination Survey (NHANES) (https://wwwn.cdc.gov/nchs/nhanes/analyticguidelines.aspx), meta-analyses combining analyses across several studies, and Mendelian Randomisation (MR) studies (see below)’.

In fact, the most striking increase in derivative studies that we have witnessed is in MR studies; last year Frontiers in Endocrinology received 552 such submissions, up from 4 in 2020. For some of the clinical specialties in our journal this represented over 15% of their total submissions. In 2023, the vast majority of MR submissions emanated from institutions in China (82.3%). The 2023 MR submission rate represented a 21.5-fold increase since 2020. Over the last 15 years there has been a general rapid rise in published MR studies: the total MR publications listed on PubMed in 2010 was just 61; by 2020 this had increased to 899; in 2023 there were 2,968 published. This represents a 48.7-fold increase in MR publications since 2010; over the same time period there was a 4.8-fold increase in bibliometric publications and a 5-fold increase in published meta-analyses. Together this has created a huge additional burden for editors and reviewers of journals, without necessarily delivering to the demands and needs of journal readers who expect the highest standards to be upheld.

MR is based on Mendel’s second law of segregation, namely that genetic variants are randomly and independently acquired at gamete formation from the parents. With this method, a genetic variant is used as a susceptibility marker for an exposure [i.e. an instrumental variable (IV)], which is then used to examine whether the exposure is causally related to an outcome, avoiding confounding, bias and reverse causality associated with observational studies, even if the exposure-outcome relationship is itself confounded (5). This can potentially prevent unwarranted further investigations based on incorrect assumptions of causality following observational studies. Initial application of MR used one-sample analyses, based on individual level data usually from a single cohort, where data was available for both the exposure and outcome combined with genetic information.

Two-sample MR analyses was subsequently developed in which gene-exposure data from one source is related to gene-outcome data from a separate source. This enabled MR to be implemented by co-analysing entirely independent genome-wide association studies (GWAS) of different traits, many of which are publicly available. Together with widely available platforms and software for undertaking MR analyses, two-sample MR can be undertaken relatively easily, without deeper understanding and expertise in genetic epidemiology. However, despite the ease with which results can be obtained, performing a rigorous MR study requires careful consideration, planning and interpretation in order to overcome assumptions and pitfalls inherent in this method. Unfortunately, this is not always adhered to, and there has been a profusion of MR publications of varying quality (6–9). That many MR papers have been published without rigorous evaluation of the underlying assumptions means that many invalid ‘causal’ associations are now in the literature; this has the potential danger of initiating inappropriate research to investigate associations identified as ‘causal’ by poorly conducted and invalid MR studies (10).

One of the most important assumptions with MR is the no pleiotropy assumption, which assumes that any relationship between the exposure IV and the outcome is mediated solely by the exposure as opposed to some other, pleiotropic pathway. MR Egger and other sensitivity methods have been developed for addressing pleiotropy, and are often included in MR papers. However, though readily automated, these are often under-powered, and other methods may need to be considered. For example, since IVs for bone mineral density (BMD) identified by GWAS are also related to BMI, multivariable MR is required to evaluate causal effects of BMD on osteoarthritis risk independently of the effects of BMI (11). Correlated pleiotropy is a particular form of pleiotropy which arises where two traits, call X and Y, are correlated, for example as a consequence of shared underlying biology (12). Whereas initial MR analysis may indicate a causal relationship of X on Y, if X and Y are correlated, an equivalent relationship is likely to be seen for Y on X. To confirm true causality, bi-directional MR analyses are required to confirm a causal relationship exists in one direction only. Power considerations are another major limitation in MR analyses. For example, if the IV used in MR analyses is only related to the exposure very weakly, this can lead to weak instrument bias (12). A related issue is that if an MR is applied to examine causal relationships with a range of outcomes, evidence thresholds need to be adjusted accordingly. Further problems can arise due to issues with measurement of the outcome; for example, incomplete phenotype information, time-variations in the exposure, measurement error, survival bias and gene-environment interactions.

As well as methodological issues, a further limitation of many MR studies is the lack of any clear justification. The relevance assumption presumes that there is good biological rationale for investigating the relationship and that the variant is strongly associated with the exposure for which it is employed as a marker. MR studies should only be performed where genuine doubt exists over the causal nature of a relationship between two variables. For example, circulating levels of vitamin D are known to be positively related to a number of health outcomes, such as BMD and fracture risk (13), which could reflect confounding given vitamin D levels are related to sun exposure and hence physical activity. MR analyses to explore these relationships are well justified, in this case finding no evidence of a causal relationship between circulating vitamin D levels and either BMD or fracture risk (12). In contrast, there is no debate as to whether premature menopause causes osteoporosis, and an MR analysis is not justifiable to establish whether a causal relationship exists in this context.

In order to address the poor quality of many published MR studies there have been several published guidelines, with helpful guides on how to conduct a MR study (14, 15), how to evaluate the instrumental variable assumptions (16, 17), how to address bias and quality (18), how to report an MR study with the STROBE-MR framework (Strengthening the Reporting of Observational Studies in Epidemiology Using MR) (19, 20), how to assess the plausibility of an MR (21) and even how to read an MR study (22). The multitude of published guidelines is an indication that undertaking an MR study that is plausible and valid is not as straightforward as may initially seem. Frontiers in Endocrinology adheres strictly to the STROBE-MR guidelines as indicated in our instructions to authors. Despite these numerous published guidelines, we are still witnessing many poor submissions and have to reject the vast majority of submitted MR studies. Other endocrine journals have had similar recent experience (23). The problems of low quality and redundant studies are not restricted to MR studies; the availability of huge databases (such as NHANES and UK Biobank) has led to many other poorly conducted epidemiology studies that offer no insight.

Initially, as few MR studies were submitted, and some editors were unfamiliar with such analyses, the rejections of the submissions to our journal were similar to that for our overall submissions. However, with the recent huge increase in MR submissions, and as editors and reviewers have become familiar with their limitations, the rejection rate has been steadily rising, now over 80% and continuing to increase. The processing of hundreds of such poor-quality submissions adds considerably to the workload of editors and reviewers. To limit over-load, we now reject submissions of MR studies that are not accompanied by a completed STROBE-MR checklist. We will similarly be imposing a condition that systematic reviews adhere to the PRISMA guidelines and include a completed checklist with submission and have a conclusion related to endocrinology. In addition, reviews including meta-analyses will have to adhere to the PRISMA extension guidelines and include a completed checklist with submission.

The profusion of mostly derivative studies, and in particular the large number of redundant manuscripts, poses a challenge to the purest ethos of open-access publishing: that it should be open, transparent, inclusive and available. Fundamentally all publications should address a biologically well-defined and reasonable question, relevant to human health and the significant health challenges the world faces, based on a sound hypothesis and that is relevant to the subject. All manuscripts should advance the knowledge-base, revealing new data or new concepts that will drive research and improve clinical practice. With the sharp increase in derivative and redundant manuscripts, together with the advent of manuscripts generated by Artificial Intelligence and ‘paper-mills’, the challenge will be to minimise the publication of redundant and meaningless manuscripts to ensure that the valuable science is not swamped. As the most cited journal with broad coverage across endocrinology and metabolism and with a commitment to open science, Frontiers in Endocrinology welcomes all credible and insightful submissions. We are, however, constantly revising procedures to filter out redundant manuscripts. With ever increasing numbers of journals open to submissions it requires editors from all journals to be diligent to prevent dilution or distortion of the endocrine literature. The drive to openness and inclusivity must not lose sight of the requirement for value, rigor, quality and expansion of true knowledge.

## Author contributions

JT: Writing – original draft, Writing – review & editing. KS: Writing – review & editing. RI: Writing – review & editing. TD: Writing – review & editing. ÅS: Writing – review & editing. ID: Writing – review & editing. JH: Writing – original draft, Writing – review & editing.
